# Effects of Presowing Pulsed Electromagnetic Treatment of Tomato Seed on Growth, Yield, and Lycopene Content

**DOI:** 10.1155/2014/369745

**Published:** 2014-07-06

**Authors:** Aspasia Efthimiadou, Nikolaos Katsenios, Anestis Karkanis, Panayiota Papastylianou, Vassilios Triantafyllidis, Ilias Travlos, Dimitrios J. Bilalis

**Affiliations:** ^1^Open University of Cyprus, P.O. Box 24801, 1304 Nicosia, Cyprus; ^2^Laboratory of Crop Production, Agricultural University of Athens, Iera Odos 75, 11855 Athens, Greece; ^3^Department of Agriculture Crop Production and Rural Environment, University of Thessaly, Fytokou Street, N. Ionia, 38466 Magnisia, Greece; ^4^Department of Business Administration of Food and Agricultural Enterprises, University of Patras, Seferi Street 2, 30100 Agrinio, Greece

## Abstract

The use of magnetic field as a presowing treatment has been adopted by researchers as a new environmental friendly technique. The aim of this study was to determine the effect of magnetic field exposure on tomato seeds covering a range of parameters such as transplanting percentage, plant height, shoot diameter, number of leaves per plant, fresh weight, dry weight, number of flowers, yield, and lycopene content. Pulsed electromagnetic field was used for 0, 5, 10, and 15 minutes as a presowing treatment of tomato seeds in a field experiment for two years. Papimi device (amplitude on the order of 12.5 mT) has been used. The use of pulsed electromagnetic field as a presowing treatment was found to enhance plant growth in tomato plants at certain duration of exposure. Magnetic field treatments and especially the exposure of 10 and 15 minutes gave the best results in all measurements, except plant height and lycopene content. Yield per plant was higher in magnetic field treatments, compared to control. MF-15 treatment yield was 80.93% higher than control treatment. Lycopene content was higher in magnetic field treatments, although values showed no statistically significant differences.

## 1. Introduction

Magnetic and electromagnetic treatments are being used in agriculture, as a noninvasive technique, to improve the germination of seeds and increase crops and yields [1]. Researchers consider that the prospect of using cheap magnetic energy to improve the properties of soil and plant growth and development may be of great practical importance [2]. Magnetic field has been found to improve food reserve utilization and help for better absorption and assimilation of nutrients by plants [3] and photosynthetic activities [4].

The choice of the investigated plants is based mainly on the importance they have. It has been found that the percent germination rates of the treated tomato seeds were accelerated about 1.1 to 2.8 times compared with that of the untreated seed, while an inhibitory effect on germination was shown in the case of the electric field more than 12 kV/cm and the exposure time more than 60 seconds [5]. Recently, it has been reported that treated tomato seeds with magnetic field by 100 gauss for 15 minutes with magnetically treated irrigated water improved vegetative growth, increased total phosphorus content of tomato leaves and total yield, and reduced pH value in soil extraction [6]. In the vegetative stage, the treatments led to a significant increase in leaf area, leaf dry weight, and specific leaf area per plant. Also, the leaf, stem, and root relative growth rates of plants derived from magnetically treated seeds were greater than those shown by the control plants. In the generative stage, leaf area per plant and relative growth rates of fruits from plants from magnetically exposed seeds were greater than those of the control plant fruits [7]. Our study examines the enhancement of magnetic field in agronomic characteristics for two years and explains how it leads to higher yield. Moreover, it uses a different type of magnetic field, to confirm the positive effect on tomato plants.

Magnetic field dose (strength and exposure duration) has been found to have strong effect on plant properties. In a recent research on garden pea seeds, of the various combinations of field strengths and exposure time, 60 mT and 180 mT for 5 min treatments yielded the better results [8]. Moreover, radish seeds were exposed to full wave rectified sinusoidal MF of different magnetic field doses and, among various combinations, 80 mT for the 5–10 min and 110 mT for the 2.5 min exposure yielded superior results [9].

Krylov and Tarakanova [10] reported that the seeds of corn and wheat with their embryonic roots oriented towards the south magnetic pole sprouted earlier than the seeds facing towards north magnetic pole. Another important enhancement of magnetic field is the improved root length, a characteristic that suggests that magnetically treated seeds can be used in practical agriculture where better root growth will enable extraction of moisture from deeper soil layers [11]. The new approach of several researchers is to provide more complete investigation of the effects of magnetic field in plants. Even more studies are directed to investigate quality characteristics, plant physiology measurements, enzyme activity, and yield production [12]. Researchers are investigating more widely the positive effects of magnetic fields spreading their findings to plant pathology. In plants derived from seeds exposed to magnetic fields, a significant delay in the appearance of first symptoms of geminivirus and early blight and a reduced infection rate of early blight were observed [7]. Pulsed electromagnetic fields showed could replace hormones in vegetative propagation of oregano, stimulating rooting process in stem cuttings [13]. Enhancements of plant characteristics with economic impact on producer's income could be the future of modern, organic, and sustainable agriculture [14]. Furthermore, the adoption of organic cultural system could reduce energy inputs [15].

The main aim of agriculture remains the yield, so the encouraging results in such measurements boost the interest of researchers to continue further. Harichand et al. [16] reported that the magnetic field treatment at 10 mT for 40 h boosted up pea height, mass, and crop yield. Lycopene is the red pigment and a major carotenoid in tomato fruit. It is a potent natural antioxidant and the focus of many breeding programs [17].

The aim of this study was to determine the effect of magnetic field exposure on tomato seeds, covering a complete range of agronomic characteristics such as transplanting percentage, plant height, shoot diameter, number of leaves per plant, fresh weight, dry weight, number of flowers, yield, and lycopene content, as an environmental friendly method.

## 2. Materials and Methods

A field experiment was established at the Agricultural University of Athens (Greece), in summer 2011 and summer 2012. A tomato (*Solanumly copersicum*) hybrid (NOXANA) was used. The duration of exposure to magnetic field was 0, 5, 10, and 15 minutes.

Tomato seeds were treated by Papimi electromagnetic field generator for 5, 10, or 15 minutes before planting. Seeds that were not treated have been used as control. Papimi device is a pulsed EMF generator (Figure 4). (Papimi model 600, Pulse Dynamics, Athens, Greece. Manufacturer characteristics: 35–80 J/pulse energy, 1 × 10^−6^ s wave duration, 35–80 × 106 W wave power, amplitude on the order of 12.5 mT, rise time 0.1 ms, fall time 10 ms, and repetitive frequency of 3 Hz.) The same device has been used in agricultural studies [13].

The tomato seedlings have been transplanted to their final position in the field after being germinated in pots filled with peat as substrate, 15 days after sowing. Fifteen days after transplanting, the measurement of successfully transplanted tomatoes has been conducted. Destructive measurements took place 100 days after transplanting (DAT). Plants were watered every day during the morning. Shoot fresh weight was measured by a precision balance and then the samples were oven-dried at 70°C for three days in order to measure the dry weight in grams per plant. Lycopene content has been measured using high performance liquid chromatography (HPLC) method as described by Hyman et al. [17].

The experiment followed a completely randomized design, with 4 main treatments (control, MF-5, MF-10, and MF-15) and 4 replications for each treatment. Every treatment was composed of 20 plants. The main factors for the statistical analysis were three (treatment, year, and replication). The experimental data were analyzed using the software Statistica [18], according to the completely randomized design. Values were compared by one-way analysis of variance (ANOVA) and mean differences were determined using the least significant difference (*LSD*) test, at the* 5%* level of significance.

## 3. Results

The use of pulsed electromagnetic field as a presowing treatment was found to enhance tomato plants in certain duration of exposure. The exposure of 10 and 15 minutes gave the best results in all measurements, except plant height and the number of leaves. The analysis of variance (ANOVA) showed that year was a nonsignificant factor, time was a significant factor, and there was no interaction between year and time of exposure (Table 1). In many measurements, the exposure of 5 minutes had a negative effect on tomato plant, where values were similar to or lower than the control. The highest response was reached at 15 min, the longest exposure tested. This means that a longer duration may give better results, a probability that must be further investigated.

**Figure 1:**
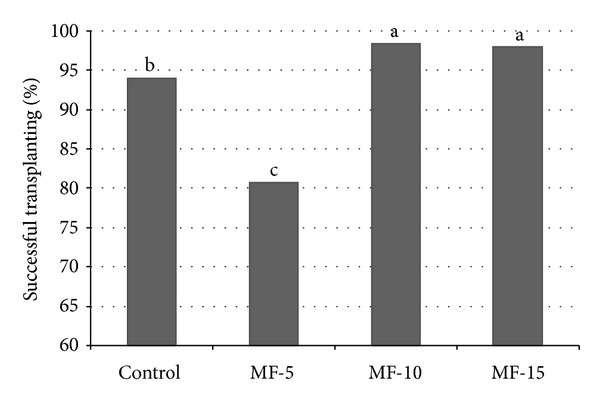
Pulsed electromagnetic field effect on percentage of successful transplanted seedlings. Seeds have been exposed for 0, 5, 10, and 15 minutes in magnetic field. Means followed by the same letter for treatments are not significantly different according to the LSD 5% test. Control: untreated seeds; MF-5, MF-10, and MF-15: seeds treated with pulsed electromagnetic field for 5 min, 10 min, and 15 min, respectively.

Pulsed electromagnetic field exposure of 10 and 15 minutes improved the percentage of successfully transplanted tomato plants (Figure 1). The highest percentage of successfully transplanted tomato plants has been recorded at* MF-10* treatment (98.41), followed by* MF-15* treatment (98.0). Both treatments gave values with statistically significant differences compared to* MF-5* and control. In this measurement* control* (94.01) was statistically significant and higher than the treatment of* MF-5* (80.70), for significant level of* 0.05*.

**Figure 2:**
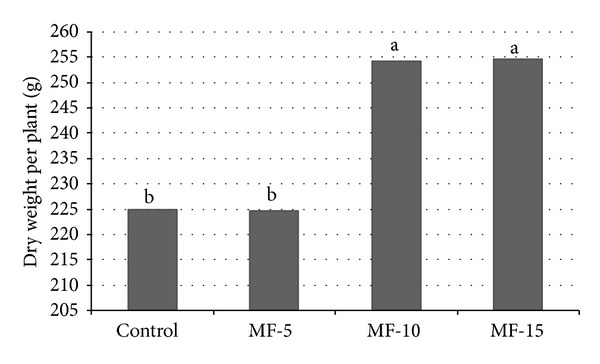
Pulsed electromagnetic field effect on dry weight per plant. Seeds have been exposed for 0, 5, 10, and 15 minutes in magnetic field. Means followed by the same letter for treatments are not significantly different according to the LSD 5% test. Control: untreated seeds; MF-5, MF-10, and MF-15: seeds treated with pulsed electromagnetic field for 5 min, 10 min, and 15 min, respectively.

Plant growth characteristics gave statistically significant differences in all measurements (Table 2, Figures 2 and 5). The highest values in plant height were measured in* control* treatment (144.4) with statistically significant differences, followed by* MF-15* (138.1) treatment.* MF-15* value was higher with statistically significant differences from* MF-10* (132.4).* MF-5* treatment (104.8) gave the lowest values with statistically significant differences.

Shoot diameter showed statistically significant differences among treatments. The highest values of shoot diameter were measured in* MF-15* treatment (17.0) where the value was statistically significant and higher than* control* (15.5) and* MF-5* (16.1) treatment, in both years.* MF-10* treatment gave the lowest values with statistically significant differences, only with* MF-15* treatment.

Number of leaves per plant was statistically significant and higher for* MF-15* (50.8), compared to all other treatments.* MF-10* treatment (47.3) gave higher values with statistically significant differences from* MF-5* (31.6) and* control* (34.5).* MF-5* gave the lowest values with statistically significant differences.

Fresh weight was significantly higher for* MF-15* (968.7), compared to all other treatments.* MF-10* treatment (964.0) gave higher values with statistically significant differences from* MF-5* (834.3) and* control* (842.9).* MF-5* gave the lowest values with statistically significant differences. Dry weight (g) was found higher in* MF-10* (254.7) and* MF-15* (254.3) treatments with statistically significant differences, compared to* MF-5* (224.7) and* control* (224.8).

The number of flowers per plant in the second week was found higher in* MF-15* treatment (16.3) with statistically significant differences. The differences between the other two magnetic field treatments (*MF-10*,* MF-5*) were not statistically significant.* Control* (14.6) treatment had no statistically significant differences compared with* MF-10* treatment.

**Figure 3:**
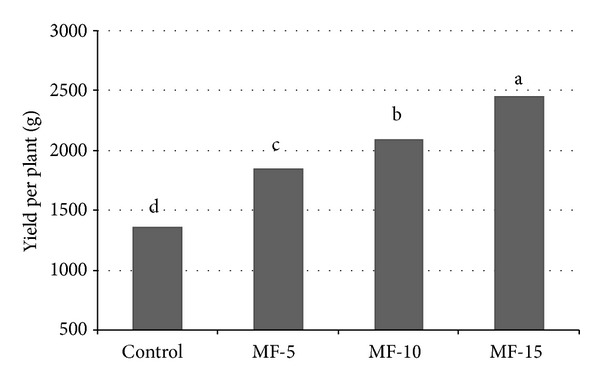
Pulsed electromagnetic field effect on yield per plant. Seeds have been exposed for 0, 5, 10, and 15 minutes in magnetic field. Means followed by the same letter for treatments are not significantly different according to the LSD 5% test. Control: untreated seeds; MF-5, MF-10, and MF-15: seeds treated with pulsed electromagnetic field for 5 min, 10 min, and 15 min, respectively.

**Figure 4:**
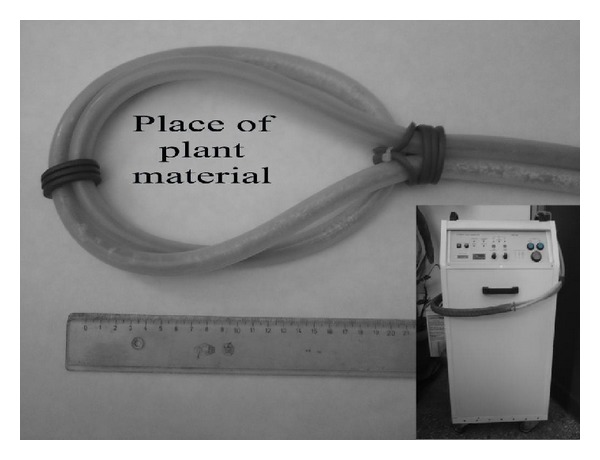
PAPIMI device and external loop.

**Figure 5:**
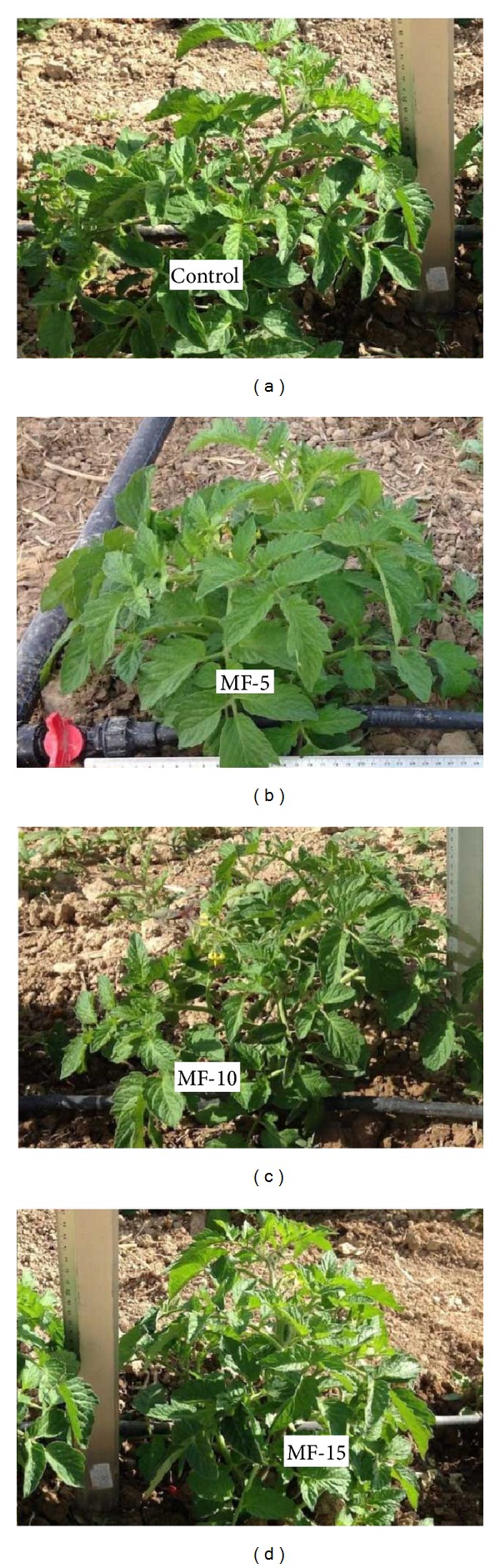
Showing the comparative effect of pulsed electromagnetic field treatment on the main agronomic plant characteristics. Control: untreated seeds; MF-5, MF-10, and MF-15: seeds treated with pulsed electromagnetic field for 5 min, 10 min, and 15 min, respectively.

The presowing application of magnetic field had positive effects on yield parameters (Figure 3). In yield per plant measurement,* MF-15* treatment (2448.8) gave the highest value, followed by* MF-10* (2082.6),* MF-5* (1845.6), and* control* (1353.5). All differences between treatments were statistically significant. Lycopene content measurements showed no statistically significant differences between treatments, although magnetic field treatments gave higher values than control. Plants derived from seeds treated for 15 minutes with magnetic field gave the highest value of lycopene content.

## 4. Discussion

The results obtained in this experiment showed a positive impact of pulsed electromagnetic field, in certain time of exposure and in tomato cultivation. Magnetic field has been found to enhance the success in transplanting, the plant growth, and the final yield. There are also indications that magnetic field could improve quality characteristics such as lycopene content.

Moon and Chung [5] found that the percent germination rates of the tomato seed treated with AC electric and magnetic fields were accelerated about 1.1–2.8 times compared with that of the untreated seeds. Presowing treatment with magnetic field in wheat seeds resulted in higher yields and gluten content [19]. In our measurements, the treatment of MF-5 gave lower percentage of successful transplanting compared to control, while MF-10 and MF-15 gave higher percentage. Inhibition of magnetic field has been found in garden pea mean emergence time, where electromagnetically treated seeds showed negative response as compared to untreated seeds [8].

Bondarenko et al. [20] used a device for magnetic field treatment in field experiments in Russia and found that in vegetable seeds the germination percentage was higher and the plant growth in early stages was higher too. Magnetic field treatment has been found to improve transpiration rate, photosynthetic rate, stomatal conductance, root growth, shoot growth, and N, P, K, Ca, and Mg percentage accumulation in early stages of cotton [21]. The increase in germination when seeds were magnetically treated could be explained by better availability and absorption of nutrients. Pulsed electromagnetic fields have been found to promote germination and improve early growth characteristics of cotton seedlings [22]. In pea, the investigation of optimal magnetic field doses showed that low magnetic field strength for the longer time of exposure and high magnetic field strength for shorter duration were found to be the most effective in enhancing the growth and yield in the pea cultivar [23].

Recently, the productivity of tomato plants using magnetic stimulated seeds or irrigation with magnetized water was investigated. The results showed that the stimulated seeds gave better results compared to the control treatments, that is, gave taller and heavier plants. Growth characteristics such as fresh weight were higher in plants grown with magnetic treatments than those grown without magnetic treatment [6]. A very important yield enhancement has been recorded in tomato plants derived from magnetic field treated seeds. The yield in cultivar Monza was 28–51% higher in early stages [24]. In a similar experiment, tomato seeds were exposed to full-wave rectified sinusoidal nonuniform magnetic fields (MFs) induced by an electromagnet at 100 mT (rms) for 10 min and at 170 mT (rms) for 3 min. The leaf, stem, and root relative growth rates of plants derived from magnetically treated seeds were greater than those shown by the control plants. At fruit maturity stage, all magnetic treatments increased significantly the mean fruit weight, the fruit yield per plant, the fruit yield per area, and the equatorial diameter of fruits in comparison with the controls [7]. In pea cultivar it has been found that the magnetic field presowing seed treatment can be used practically to enhance the growth and yield [23].

Lycopene content values were higher in magnetic field treatments, although without statistically significant differences. Higher time of exposure, for example, 25 minutes, can be used for further evaluation. Lycopene as a carotenoid pigment is a very important quality characteristic for tomatoes. In date palm it has been found that pigments content (chlorophyll a, chlorophyll b, carotenoids, and total pigments) was significantly increased under static magnetic field, while the highest measurements were recorded at 100 mT, after 360 min of exposure [25]. Chlorophyll a and carotenoids were more affected than chlorophyll b in date palm seedlings.

## 5. Conclusion

These results indicate that the application of magnetic field in tomato seeds can be an ecofriendly practice that improves plant characteristics in all stages, from germination to final yields. Magnetic field treatment, in certain times of exposure, improved shoot diameter, number of leaves per plant, fresh and dry weight, number of flowers, and yield per plant. These studies provide a holistic approach of an agricultural cultivation that can lead to the comprehension of the exact mechanism of magnetic field effect on plant tissues and lead to the appropriate application of the magnetic fields. The determination of the optimal duration of exposure, the type of magnetic field used, and the effect of magnetic field on quality characteristics are some major factors that deserve further investigation.

## Figures and Tables

**(a):** 

	Percentage of successfully transplanted tomatoes (%)	Plant height (cm)	Shoot diameter (mm)	Number of leaves per plant	Fresh weight (g)
Year	1.31^ns^	0.008^ns^	8.90^ns^	10.02^ns^	10.91^ns^
Time	365.22^∗∗∗^	1869.73^∗∗∗^	2.49^ns^	693.80^∗∗∗^	1899.14^∗∗∗^
Year ∗ time	0.90^ns^	0.04^ns^	2.87^ns^	0.50^ns^	0.50^ns^

**(b):** 

	Dry weight (g)	Number of flowers in the second week	Yield (g per plant)	Lycopene content (*μ*g/100 g fresh weight)
Year	0.03^ns^	11.79^ns^	6.20^ns^	0.34^ns^
Time	133.17^∗∗∗^	6.33^∗∗∗^	3924.19^∗∗∗^	0.34^ns^
Year ∗ time	0.22^ns^	0.43^ns^	0.84^ns^	0.10^ns^

^*∗*,*∗∗*,*∗∗∗*^Significance at 0.05, 0.01, and 0.001; ns: not significant.

**Table 2:** Mean values of main plant characteristics and quality measurements.

	Plant height (cm)	Shoot diameter (mm)	Number of leaves per plant	Fresh weight (g)	Number of flowers in the second week	Lycopene content (*μ*g/100 g fresh weight)
Control	144.4^a^	15.5^b^	34.5^d^	842.9^d^	14.6^c^	6083^a^
MF-5	104.8^d^	16.1^b^	31.6^c^	834.3^c^	15.2^b^	6130^a^
MF-10	132.4^c^	16.3^ab^	47.3^b^	964.0^b^	15.0^bc^	6122^a^
MF-15	138.1^b^	17.0^a^	50.8^a^	968.7^a^	16.3^a^	6214^a^

Control: untreated seeds; MF-5, M F-10, and MF-15: seeds treated with pulsed electromagnetic field for 5 min, 10 min, and 15 min, respectively.

^a,b,c,d^Means followed by the same letter for treatments are not significantly different according to the least significant difference (LSD) test.
