# Corrigendum to “Effects of Presowing Pulsed Electromagnetic Treatment of Tomato Seed on Growth, Yield, and Lycopene Content”

**DOI:** 10.1155/2016/9174782

**Published:** 2016-07-28

**Authors:** Aspasia Efthimiadou, Nikolaos Katsenios, Anestis Karkanis, Panayiota Papastylianou, Vassilios Triantafyllidis, Ilias Travlos, Dimitrios J. Bilalis

**Affiliations:** ^1^Open University of Cyprus, P.O. Box 24801, 1304 Nicosia, Cyprus; ^2^Laboratory of Crop Production, Agricultural University of Athens, Iera Odos 75, 11855 Athens, Greece; ^3^Department of Agriculture Crop Production and Rural Environment, University of Thessaly, Fytokou Street, N. Ionia, 38466 Magnisia, Greece; ^4^Department of Business Administration of Food and Agricultural Enterprises, University of Patras, Seferi Street 2, 30100 Agrinio, Greece

 In the article titled “Effects of Presowing Pulsed Electromagnetic Treatment of Tomato Seed on Growth, Yield, and Lycopene Content”, [1] Figure 4 showing the PAPIMI device was already published in a previous article by the authors, which was cited as reference 13. Figure 4 should be replaced with the following figure.

## Figures and Tables

**Figure 4:**
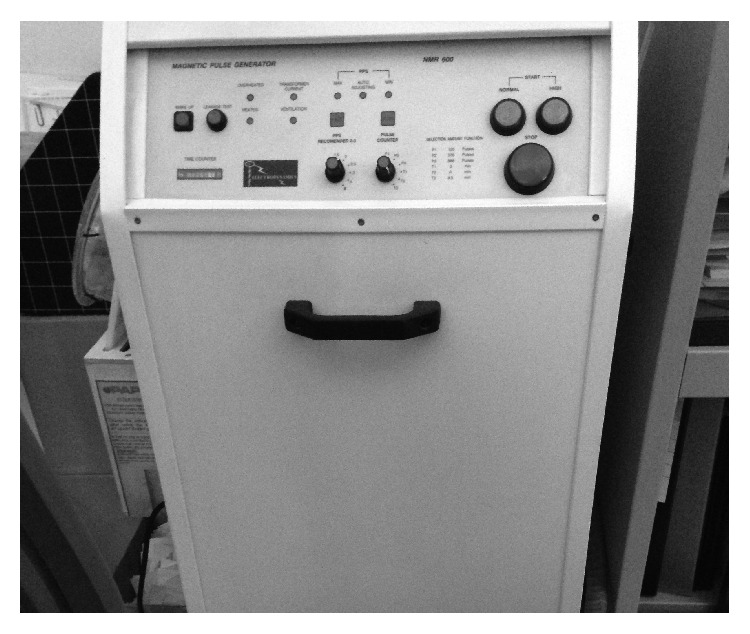
PAPIMI device.
